# Alterations in fatty acid metabolism and sirtuin signaling characterize early type‐2 diabetic hearts of fructose‐fed rats

**DOI:** 10.14814/phy2.13388

**Published:** 2017-08-22

**Authors:** Phing‐How Lou, Eliana Lucchinetti, Katrina Y. Scott, Yiming Huang, Manoj Gandhi, Martin Hersberger, Alexander S. Clanachan, Hélène Lemieux, Michael Zaugg

**Affiliations:** ^1^ Department of Pharmacology University of Alberta Edmonton Alberta Canada; ^2^ Department of Anesthesiology and Pain Medicine University of Alberta Edmonton Alberta Canada; ^3^ Division of Clinical Chemistry and Biochemistry University Children's Hospital Zürich Zurich Switzerland; ^4^ Faculty Saint‐Jean University of Alberta Edmonton Alberta Canada

**Keywords:** Cardiolipin, fatty acid metabolism, fructose, insulin resistance, mitochondria, oxidative stress, sirtuins, type‐2 diabetes mellitus

## Abstract

Despite the fact that skeletal muscle insulin resistance is the hallmark of type‐2 diabetes mellitus (T2DM), inflexibility in substrate energy metabolism has been observed in other tissues such as liver, adipose tissue, and heart. In the heart, structural and functional changes ultimately lead to diabetic cardiomyopathy. However, little is known about the early biochemical changes that cause cardiac metabolic dysregulation and dysfunction. We used a dietary model of fructose‐induced T2DM (10% fructose in drinking water for 6 weeks) to study cardiac fatty acid metabolism in early T2DM and related signaling events in order to better understand mechanisms of disease. In early type‐2 diabetic hearts, flux through the fatty acid oxidation pathway was increased as a result of increased cellular uptake (CD36), mitochondrial uptake (CPT1B), as well as increased *β*‐hydroxyacyl‐CoA dehydrogenase and medium‐chain acyl‐CoA dehydrogenase activities, despite reduced mitochondrial mass. Long‐chain acyl‐CoA dehydrogenase activity was slightly decreased, resulting in the accumulation of long‐chain acylcarnitine species. Cardiac function and overall mitochondrial respiration were unaffected. However, evidence of oxidative stress and subtle changes in cardiolipin content and composition were found in early type‐2 diabetic mitochondria. Finally, we observed decreased activity of SIRT1, a pivotal regulator of fatty acid metabolism, despite increased protein levels. This indicates that the heart is no longer capable of further increasing its capacity for fatty acid oxidation. Along with increased oxidative stress, this may represent one of the earliest signs of dysfunction that will ultimately lead to inflammation and remodeling in the diabetic heart.

## Introduction

Type‐2 diabetes mellitus (T2DM) is the most prevalent form of diabetes. Its pathogenesis is multi‐factorial and is typically characterized by a combination of insulin resistance (impaired insulin‐mediated glucose disposal) and aberrant lipid metabolism, which consequently leads to accumulation of triglycerides, mitochondrial dysfunction, oxidative stress, and inflammation (Boudina and Abel 2010). The clustering of these conditions is often referred to as the metabolic syndrome, the manifestation of which is a major risk factor for cardiovascular morbidity and mortality.

Consumption of fructose (high‐fructose corn syrup) and calorie‐dense (sugar‐ and/or fat‐enriched) food and beverages is considered to be a main contributing factor to the current diabetic and obesity epidemic. Due to the lipogenic nature of fructose (Basciano et al. 2005), fructose feeding resembles the nutritional overload seen with high‐fat diets. It is known to induce metabolic changes that are typical of T2DM in rodents and humans alike. The fructose‐fed rat, a model of the metabolic syndrome originally described by Zavaroni et al. (1980), exhibits fasting hyperglycemia, hyperinsulinemia, insulin resistance, hypertriglyceridemia, and arterial hypertension (Dai and McNeill 1995; Thorburn et al. 1989; Tran et al. 2009). The effects of fructose‐rich diets, particularly on insulin sensitivity, are time‐ and tissue‐dependent (Dai and McNeill 1995; Delbosc et al. 2005; Polakof et al. 2016) and appear to be reversible, at least if feeding does not exceed 12 weeks (Dai and McNeill 1995). Rats receiving drinking water enriched with 10% fructose for a period of 6 weeks are insulin resistant and represent a valuable model for early stage T2DM, which we have robustly replicated in all of our previous studies (Lou et al. 2014, 2015; Warren et al. 2014). Our experience with 6‐week fructose feeding in Sprague‐Dawley rats showed that insulin‐stimulated glucose uptake was decreased in the heart without overt signs of cardiac dysfunction (Lou et al. 2015). On the other hand, the skeletal muscle revealed fiber‐specific (glycolytic extensor digitorum longus vs. oxidative soleus) differences in their respective onset of metabolic derangements and oxidative stress, despite an overall conserved mitochondrial function and capacity (Warren et al. 2014). In this study, we aimed to characterize and elucidate the metabolic (mal)adaptations as well as oxidative stress that occur in fructose‐induced type‐2 diabetic hearts at early stages of the disease. These events may represent common factors leading to heart failure, and may be integral parts of the primary mechanisms underlying diabetic cardiomyopathy.

## Materials and Methods

### Chemicals

All chemicals were from Sigma–Aldrich (Oakville, ON, Canada), unless otherwise stated.

### Animals

All animal experiments were conducted in accordance to the Guide for the Care and Use of Laboratory Animals published by the National Institutes of Health (NIH publication, 8th ed., 2011) and was approved by the University of Alberta Animal Policy and Welfare Committee. Male Sprague‐Dawley rats at 6 weeks of age were housed and maintained on a 12‐h/12‐h light/dark cycle and allowed free access to standard rodent chow and either water ad libitum (control group) or 10% fructose in the drinking water (fructose‐fed group) for 6 weeks, as previously done (Warren et al. 2014). Thereafter, glucose tolerance test and insulin sensitivity tests were performed by serial measurements of blood glucose after intraperitoneal injection of d‐glucose (2 g/kg body weight) or subcutaneous injection of recombinant human insulin (0.5 U/kg body weight; Novolin®ge Toronto, Novo Nordisk Canada Inc, Mississauga ON, Canada).

### Echocardiography

Echocardiography was performed in control and fructose‐fed rats after 6 weeks of feeding. Cardiac function was assessed using echocardiography under light isoflurane anesthesia, using a Vevo 770 high‐resolution imaging system equipped with a 30 MHz transducer (RMV‐707B, VisualSonics, Toronto, Canada).

### Mitochondrial respirometric measurements

The whole left ventricle of the rat heart was excised for mitochondrial isolation, using a modified method of King et al. (2007). The final mitochondrial suspension was immediately used for measurement of mitochondrial respiratory parameters. Mitochondrial respirometry was measured using the Oroboros Oxygraph 2K system (Oroboros, Innsbruck, Austria) at 37°C, in a similar manner as previously reported (Warren et al. 2014). The following substrates were used to evaluate oxidative phosphorylation driven by fatty acid oxidation substrates: palmitoylcarnitine (2.5–15 *μ*mol L^−1^) + malate (4 mmol L^−1^), palmitoyl‐CoA (20 *μ*mol L^−1^) + carnitine (1.25 mmol L^−1^) + malate (2 mmol L^−1^), octanoylcarnitine (330 *μ*mol L^−1^) + malate (2 mmol L^−1^), acetylcarnitine (5 mmol L^−1^) + malate (2 mmol L^−1^); state III (ADP‐stimulated, 5 mmol L^−1^) respiration or state IV (oligomycin‐inhibited, 2 *μ*g mL^−1^) respiration. High concentrations of palmitoylcarnitine potentially have a detergent effect on the mitochondrial membranes, which suppresses mitochondrial respiration (Cho and Proulx 1969), and so a palmitoylcarnitine titration was initially performed to identify the optimal concentration for maximal respiration. A single addition of this concentration was then applied to the palmitoylcarnitine/malate protocol. Measurements with complex‐specific substrates were also performed: pyruvate (5 mmol L^−1^) + malate (2 mmol L^−1^), glutamate (40 mmol L^−1^) + malate (8 mmol L^−1^), succinate (10 mmol L^−1^) + rotenone (0.5 *μ*mol L^−1^), and ascorbate (2 mmol L^−1^) + *N,N,N′,N′*‐tetramethyl‐i‐phenylenediamine (TMPD, 0.5 mmol L^−1^). Additional measurements of mitochondrial respiration were made with cytochrome *c* (10 *μ*mol L^−1^), in the presence and absence of pyruvate, malate, and ADP. A cytochrome effect of <10% validates the integrity of the outer mitochondrial membrane (Kuznetsov et al. 2004). Final respirometry data were expressed as pmol O/(s *μ*g) mitochondria.

### Enzymatic activity assays

Electron transport complex activities were determined as specific donor‐acceptor oxidoreductase activities using mitochondrial samples that had undergone 3 cycles of freeze/thaw in hypotonic buffer (25 mmol L^−1^ potassium phosphate/5 mmol L^−1^ MgCl_2_, pH 7.2) (Kirby et al. 2007). Complex I activity was measured as rotenone‐sensitive oxidation of NADH (340 nm) with 65 *μ*mol L^−1^ ubiquinone as substrate (50 mmol L^−1^ potassium phosphate pH 7.5, 2.5 mg mL^−1^ defatted BSA, 130 *μ*mol L^−1^ NADH, 2 mmol L^−1^ KCN, 10 *μ*g mL^−1^ antimycin A, 2 *μ*g mL^−1^ rotenone). Complex II activity was assessed as malonate‐sensitive reduction in 2,6‐dichlorophenolindophenol (DCPIP, 600 nm) with 20 mmol L^−1^ succinate as substrate (25 mmol L^−1^ potassium phosphate pH 7.5, 1 mg mL^−1^ defatted BSA, 50 *μ*mol L^−1^ DCPIP, 2 mmol L^−1^ KCN, 10 *μ*g mL^−1^ antimycin A, 2 *μ*g mL^−1^ rotenone, 10 mmol L^−1^ malonate, 65 *μ*mol L^−1^ ubiquinone). Complex III activity was determined as antimycin A‐sensitive reduction in cytochrome *c* (550 nm) with 200 *μ*mol L^−1^ decylubiquinol as substrate (25 mmol L^−1^ potassium phosphate pH 7.5, 1 mmol L^−1^ n‐dodecyl‐b‐D‐maltoside, 2 *μ*g mL^−1^ rotenone, 1 mmol L^−1^ KCN, 100 *μ*mol L^−1^ EDTA, 75 *μ*mol L^−1^ oxidized cytochrome *c*, 10 *μ*g mL^−1^ antimycin A). Complex IV activity was measured as the oxidation of reduced cytochrome *c* (550 nm) under conditions: 50 mmol L^−1^ potassium phosphate pH 7.0, 450 *μ*mol L^−1^ *n*‐dodecyl‐*b*‐D‐maltoside, 1 mmol L^−1^ KCN, 100 *μ*mol L^−1^ reduced cytochrome *c*. All assays were performed at 30°C. Carnitine palmitoyltransferase (CPT) activity was measured in mitochondrial samples using 5,5′‐dithiobis‐(2‐nitrobenzoic acid) (DTNB) to monitor the release of reduced CoA liberated from palmitoyl‐CoA by CPT at 412 nm, as previously described (Lou et al. 2014). *β*‐hydroxyacyl‐CoA dehydrogenase (*β*‐HCAD) activity of mitochondrial isolates was measured in buffer containing 97 mmol L^−1^ potassium phosphate, and 100 *μ*mol L^−1^ NADH, pH 7.3. The reaction was started with 90 *μ*mol L^−1^ S‐acetoacetyl‐CoA and the oxidation of NADH was monitored at 430 nm for 5 min. Acyl‐CoA dehydrogenase activities were assayed in mitochondrial isolates by monitoring the bleaching of DCPIP at 600 nm, using either octanoyl‐CoA (medium‐chain) or palmitoyl‐CoA (long‐chain) as substrate (Ikeda et al. 1983). Aldehyde dehydrogenase‐2 (ALDH2) activity was determined from mitochondrial isolates, by monitoring the reduction in NAD^+^ to NADH (340 nm) at room temperature, in 50 mmol L^−1^ sodium pyrophosphate pH 9.5, 10 mmol L^−1^ acetaldehyde, and 2.5 mmol L^−1^ NAD^+^. Aconitase activity was measured in mitochondria that were isolated in the presence of citrate and MnCl_2_, to limit aconitase inactivation during sample preparation and storage (Gardner et al. 1994; Bulteau et al. 2003). Aconitase activity was assayed as previously outlined (Lou et al. 2014). Oxalomalate (2 mmol L^−1^, Cayman), a competitive inhibitor of aconitase, was included in a separate set of samples to ensure assay specificity. The activity of citrate synthase (CS), was spectrophotometrically monitored in tissue homogenates by following the formation of thionitrobenzoate (TNB) at 412 nm under the following conditions: 100 mmol L^−1^ Tris‐HCl (pH 8.1), 0.1 mmol L^−1^ DTNB, 0.5 mmol L^−1^ oxaloacetate, 0.31 mmol L^−1^ acetyl CoA, 0.25% Triton X‐100, as previously described (Srere 1969). NF‐kB (p65) DNA‐binding activity was assayed using nuclear fraction prepared as recommended by the manufacturer (Thermo Scientific). The activities of pyruvate dehydrogenase (PDH), SIRT1, and SIRT3 activities were measured using kits purchased from Abcam. Superoxide dismutase (SOD) activity was measured according to manufacturer's specifications (BioVision Incorporated, CA). The following measurements were performed according to respective manufacturer's instructions: glutathione (BioVision), malonyl‐CoA (MyBiosource), NAD^+^/NADH and NADP^+^/NADPH (Abcam).

### Subcellular fractionations

Subcellular fractionations of mitochondria, nuclear and sarcolemma by differential centrifugation were performed according to the methods of Cox and Emili (2006). Protein concentrations were quantified by Bradford assay.

### Immunoblotting

Total tissue homogenates were prepared using lysis buffer (50 mmol L^−1^ Tris pH 8.0, 150 mmol L^−1^ NaCl, 1% NP‐40, 0.1% SDS, 0.5% sodium deoxycholate) supplemented with the appropriate protease and phosphatase inhibitors. Protease and phosphatase inhibitors were also included in the solutions used in the preparation of subcellular fractionations for immunoblotting. All prepared tissue homogenates or subcellular fractions were mixed in 5X SDS loading buffer for Western blot analyses. Equal amounts of protein samples were loaded, separated by SDS‐PAGE and transferred to nitrocellulose membranes for immunoblotting with indicated antibodies. Immuno‐reactivity was visualized by an ECL reaction (GE HealthCare), and its intensity was quantified using ImageJ software (http://rsbweb.nih.gov/ij/). The following primary antibodies were sourced from (1) Cell Signaling Technology (Danvers, MA): pan‐acetyl‐lysine (#9441), phospho‐AMPK*α* (Thr172; #2531), AMPK*α* (#2532), lamin A/C (#4777), Na^+^/K^+^‐ATPase (#3010), (2) Abcam Inc. (Cambridge, MA): CD36 (#ab133625), COX IV (#ab14744), CPT1B (#ab104662), PDK4 (#ab89295), PGC1*α* (#ab54481), PPAR*α* (#ab8934), *α*‐tubulin (#ab7291); (3) Santa Cruz Biotechnology Inc. (Dallas, TX): SIRT1 (#sc‐15404), SIRT3 (#sc‐99143); (4) Millipore (San Diego, CA): HNE‐Michael adducts (#393207); (5) Acris Antibodies (San Diego, CA): glutathione peroxidase I (GPx1; #AP32028PU‐N); (6) Thermo Scientific (Rockford): thioredoxin reductase 2 (TrxR2; #PA1‐20940).

### Transmission electron microscopy

Left ventricular biopsies were prefixed in 2.5% glutaraldehyde in 0.1 mol L^−1^ sodium cacodylate buffer, postfixed in 2% osmium tetroxide (OsO_4_) in 0.1 mol L^−1^ sodium cacodylate buffer, dehydrated in ethyl alcohol, and embedded in epoxy resin. Ultra thin‐sections (60 nm) were cut using an ultramicrotome (Leica UC7, Leica Microsystems Inc., ON, Canada), stained with 4% uranyl acetate and Reinold's lead citrate and imaged under a Hitachi H‐7650 transmission electron microscope at 80 kV equipped with a 16 megapixel EMCCD camera (XR111, Advanced Microscopy Technique, MA). Photoshop (Adobe) was used to adjust contrast and brightness of transmission electron micrographs, trace mitochondrial boundaries on the micrographs, and color the traced areas. The colored areas which represent mitochondrial mass were quantified by ImageJ software.

### Mass spectrometry for cardiolipin analysis and acylcarnitine profiling

Lipids were extracted from cardiac tissue samples using a mixed methanol‐chloroform (3:1, v/v) solvent twice. Lipid extracts were dried under a nitrogen gas flow at 30°C. The dried residues were dissolved in methanol (12.5 *μ*L per mg of the raw material). UPLC‐MS runs were carried out on a Waters Acquity UPLC system coupled to a Thermo Scientific LTQ‐Orbitrap Fusion Fourier transform (FT) mass spectrometer with positive‐ion (+) electrospray ionization. The MS instrument was operated in the (+) ion survey scan mode with Fourier transform (FT) MS detection (a 120,000‐mass resolution at *m/z* 400) for separation and detection of cardiolipin (CL) species. Separation of cardiolipins from other lipids was performed on a silica‐based hydrophilic interaction liquid chromatography (HILIC) column and with methanol – ammonium acetate buffer as the mobile phase for binary solvent gradient elution, using a custom‐developed procedure. Tetramyristoyl‐cardiolipin (CL[56:0]) and tetraoleoyl‐cardiolipin (CL[72:4]) were purchased from Avanti Polar Lipids, Inc. (Alabaster) and used as internal standard (IS). Tissue levels of acylcarnitine species were measured using electrospray ionization tandem mass spectrometry. Acylcarnitines were extracted from heart tissue with methanol and quantified using eight isotopically labeled internal standards (Cambridge Isotopes Laboratories, Andover, MA). Precursor ions of *m/z* 85 in the mass range of *m/z* 150–450 were acquired on a PE SCIEX API 365 LC‐ESI‐MS/MS instrument (Applied Biosystems, Foster City, CA).

### Statistical analysis

SigmaPlot 13.0 (Systat Software, San Jose, CA) was used for statistical analyses. Results are presented as mean ± SE for the indicated number of independent heart experiments. Differences between control and fructose‐fed rats were evaluated by Student's *t*‐test or Mann–Whitney rank sum tests, as determined by data distribution. Differences are considered significant when *P* < 0.05.

## Results

### Insulin resistance but normal cardiac function in hearts of early type‐2 diabetic fructose‐fed rats

**Figure 1:**
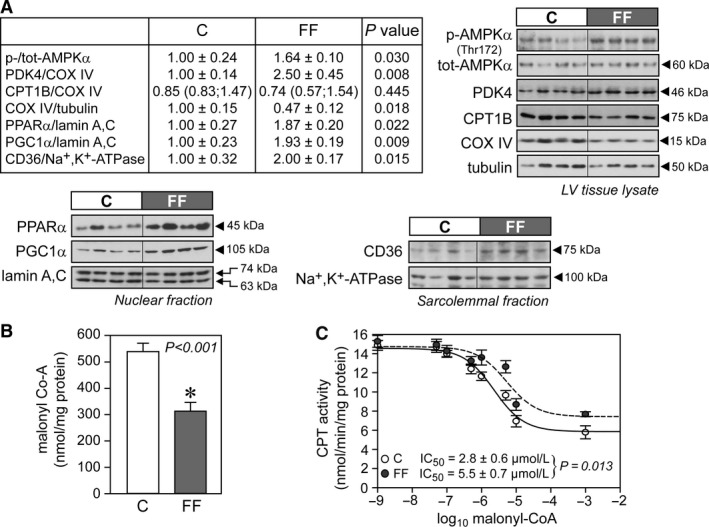
Increased cellular and mitochondrial fatty acid uptake in hearts of fructose‐fed rats. (A) Representative immunoblots and densitometric quantification of cellular AMPK, PDK4, CPT1B, and COX IV, nuclear PPAR *α* and PGC1*α*, and plasma membrane CD36 (*n* = 6–7/group). (B) Malonyl‐CoA content from total cardiac tissue samples (*n* = 7/group). (C) Semi‐logarithmic plot of CPT activity at graded malonyl‐CoA concentrations (0, 0.05, 0.1, 0.5, 1, 5, 10, and 1000 *μ*mol L^−1^; *n* = 7/group), with averaged IC_50_ values calculated from independent plots for each heart in each independent treatment group. Data were normalized to mitochondrial protein concentration. Results are mean ± SE. C, control rats; FF, fructose‐fed rats; AMPK, 5′ AMP‐activated protein kinase; CD36, fatty acid translocase; COX IV, complex IV (cytochrome *c* oxidase); CPT, carnitine palmitoyltransferase; PDK4, pyruvate dehydrogenase kinase 4; PPAR *α*, peroxisome proliferator‐activated receptor alpha; PGC1*α*, peroxisome proliferator‐activated receptor gamma coactivator 1‐alpha.

After 6 weeks of fructose feeding, rats were hyperglycemic, hyperinsulinemic, and showed delayed whole body glucose clearance (Table 1). The hearts of fructose‐fed rats were insulin resistant as evidenced by the reduction in pyruvate dehydrogenase (PDH) activity (Table 2), the rate‐limiting enzyme for glucose oxidation, and increased expression of PDH kinase 4 (PDK4) (Fig. 1A). These results are consistent with our previous experiments showing decreased activation of downstream signaling (such as Akt) upon insulin stimulation and significant reduction in insulin‐stimulated glucose uptake (Lou et al. 2015). At this early stage of insulin resistance, there were no signs of cardiac dysfunction (Table 3) or of cardiac hypertrophy (no increase in atrial natriuretic peptide and *α*‐skeletal muscle actin protein levels, data not shown), confirming previous observations (Calabro et al. 2016).

**Table 1:** Effects of fructose feeding on metabolic parameters

	C	FF	*P* value
Plasma fasting glucose (mg dL^−1^)	95 (78; 97)	124 (123; 136)	**0.008**
Plasma fasting insulin (ng mL^−1^)	1.066 ± 0.21	2.69 ± 0.17	**0.0003**
QUICKI	0.295 ± 0.007	0.250 ± 0.002	**0.0004**
Plasma triglycerides (mg mL^−1^)	62 (58; 90)	341 (279; 487)	**<0.001**
GTT‐AUC (mmol L^−1^ min^−1^)	1053 ± 63	1569 ± 40	**0.0001**
IST‐AUC (mmol L^−1^ min^−1^)	452 (439; 457)	717 (686; 802)	**0.008**

Data are presented as mean ± SE or median (25th; 75th percentile). *n* = 5 (all parameters, except plasma triglycerides), *n* = 10 (triglycerides). Bold indicates the results significant at the 0.05 level.

C, age‐matched control rats (fed standard chow and water); FF, rats fed standard chow and 10% fructose added to the drinking water; AUC, area‐under‐the‐curve; GTT, glucose tolerance test; IST, insulin sensitivity test; QUICKI, quantitative insulin sensitivity check index.

**Table 2:** Enzymatic activities

	C	FF	*n*	*P* value
Mitochondrial complex				
Complex I (*μ*mol min^−1^ mg^−1^)	0.58 ± 0.03	0.71 ± 0.04	8	**0.021**
Complex II (*μ*mol min^−1^ mg^−1^)	0.17 ± 0.01	0.13 ± 0.01	8	0.052
Complex III (*μ*mol min^−1^ mg^−1^)	4.00 ± 0.31	4.38 ± 0.32	8	0.410
Complex IV (*μ*mol min^−1^ mg^−1^)	6.31 ± 0.50	5.27 ± 0.26	8	0.085
CPT activity				
CPT total (*μ*mol min^−1^ g^−1^)	13.14 ± 0.55	14.45 ± 1.31	7	0.372
CPT 2 (*μ*mol min^−1^ g^−1^)	7.93 ± 0.72	11.75 ± 1.11	7	**0.013**
CPT 1 (*μ*mol min^−1^ g^−1^)	5.21 ± 0.18	2.71 ± 0.60	7	**0.002**
*β*‐Oxidation				
LCAD (nmol min^−1^ mg^−1^)	9.93 ± 0.42	8.27 ± 0.30	6	**0.009**
MCAD (nmol min^−1^ mg^−1^)	3.15 ± 0.23	4.35 ± 0.18	6	**0.002**
*β*‐HCAD (nmol min^−1^ mg^−1^)	3.18 ± 0.34	4.31 ± 0.32	8	**0.029**
Other				
Aconitase (*μ*mol min^−1^ mg^−1^)	96.4 ± 9.4	46.1 ± 4.8	8	**0.0005**
ALDH2 (nmol min^−1^ mg^−1^)	626 ± 83	263 ± 42	6	**0.003**
Citrate Synthase (*μ*mol min^−1^ mg^−1^)	0.96 ± 0.03	0.81 ± 0.02	6	**0.003**
PDH activity (mOD min^−1^ CS^−1^)	100.9 ± 4.6	83.4 ± 4.4	7	**0.017**
SOD, mitochondrial (U mg^−1^)	3.87 ± 0.54	3.07 ± 0.24	6	0.244
SOD, cytosolic (U mg^−1^)	203 ± 13	228 ± 21	6	0.339

Data are presented as mean ± SE. Bold indicates the results significant at the 0.05 level.

C, age‐matched control rats (fed standard chow and water); FF, rats fed standard chow and 10% fructose added to the drinking water; *β*‐HCAD, *β*‐hydroxyacyl‐CoA dehydrogenase; MCAD, acyl‐CoA dehydrogenase, medium chain; LCAD, acyl‐CoA dehydrogenase, long chain; ALDH2, aldehyde dehydrogenase‐2; CPT, carnitine palmitoyltransferase; CS, citrate synthase; PDH, pyruvate dehydrogenase; SOD, superoxide dismutase.

**Table 3:** Echocardiography

	C	FF	*P* value
M‐mode left ventricular internal diameter trace (LV Mass Protocol)			
HR (min^−1^)	344 ± 13	380 ± 24	0.205
LVID;d (mm)	7.9 ± 0.5	7.7 ± 0.5	0.761
LVID;s (mm)	3.6 (2.8; 5.3)	3.7 (3.1; 4.0)	0.905
Vol;d (mm^3^)	342 ± 45	320 ± 40	0.735
Vol;s (mm^3^)	78 ± 24	56 ± 9	0.485
SV (mm^3^)	265 ± 21	264 ± 34	0.987
EF (%)	82 (70; 88)	82 (79; 86)	0.905
FS (%)	52 (41; 60)	52 (50; 57)	0.905
CO (mm^3^/min)	91 ± 9	100 ± 14	0.592
Cardiac geometry			
IVS;d (mm)	2.2 (2.1; 2.2)	2.1 (2.1; 2.5)	0.548
IVS;s (mm)	3.6 (3.4; 3.6)	3.5 (3.5; 4.2)	1.000
LVPW;d (mm)	2.1 ± 0.1	2.4 ± 0.1	0.076
LVPW;s (mm)	3.4 ± 0.2	3.7 ± 0.2	0.431
LVID;d (mm)	7.8 ± 0.4	8.0 ± 0.4	0.829
LVID;s (mm)	4.1 ± 0.5	4.0 ± 0.3	0.855
LV Mass (g)	1103 ± 97	1278 ± 127	0.305
Mitral tissue doppler measures			
E/A	1.31 ± 0.10	1.26 ± 0.09	0.704
DS (mm/sec^2^)	24 (17; 30)	30 (17; 50)	0.548
IVRT (msec)	27.0 ± 0.9	26.4 ± 1.8	0.375
IVCT (msec)	19.3 ± 1.0	19.5 ± 1.3	0.895
ET (msec)	56.3 ± 2.3	58.0 ± 3.4	0.344
TEI index	0.83 ± 0.03	0.80 ± 0.05	0.637
E’/A’	0.98 ± 0.06	1.06 ± 0.02	0.196
E/E’	18.3 ± 1.8	22.6 ± 5.2	0.461
S’	54.5 ± 2.0	48.0 ± 2.2	0.060
Pulmonary venous flow profile			
s‐wave (mm/sec)	267 ± 16	257 ± 12	0.624
d‐wave (mm/sec)	520 ± 23	593 ± 66	0.377
a‐wave (mm/sec)	143 ± 18	199 ± 27	0.152
PVad (msec)	17.8 ± 0.8	20.2 ± 2.1	0.366
Other			
Desc AoV (mm/sec)	1048 ± 31	853 ± 48	**0.026**
Asc AoV (mm/sec)	1203 (1115; 1358)	1061 (1018; 1365)	0.250

Data are presented as mean ± SE or median (25th percentile; 75th percentile); *n* = 5 per group. Bold indicates the results significant at the 0.05 level.

d, diastole; s, systole; HR, heart rate; LVID, left ventricular interrior diameter; Vol, volume; SV, stroke volume; EF, ejection fraction; FS, fractional shortening; CO, cardiac output; IVS, interventricular septum; LVPW, left ventricle posterior wall thickness; LVID, left ventricular interrior diameter; E, peak early diastolic left ventricular (LV) inflow velocity; A, late diastolic LV inflow velocity; E/A, ratio of early to late LV diastolic filling velocities; DS, deceleration slope of the E wave; IVRT, isovolumic relaxation time; IVCT, isovolumic contraction time, ET, ejection time, TEI index, myocardial performance index, calculated as [IVCT + IVRT]/ET; E’, peak early diastolic velocity at the mitral annulus; A’, late diastolic LV inflow velocity at the mitral annulus; S’, systolic velocity at the mitral annulus; PVad, pulmonary venous a‐wave duration; Desc AoV, descending aorta pulse wave velocity; Asc AoV, ascending aorta pulse wave velocity.

### Mitochondrial fatty acid oxidation capacity is increased in hearts of early type‐2 diabetic fructose‐fed rats

Levels of nuclear PPAR*α* and PGC1*α* that transactivate multiple genes involved in myocardial fatty acid utilization were enhanced in hearts of fructose‐fed rats (Fig. 1A). In addition, the abundance of CD36 and AMPK activity (p‐AMPK), which are involved in the cellular uptake of fatty acids, were also increased (Fig. 1A). In contrast, the activity of CPT1, a key determinant of mitochondrial fatty acid uptake capacity, was reduced in hearts of fructose‐fed rats (Table 2). However, the myocardial concentration of malonyl‐CoA (a potent inhibitor of CPT1) was lower in hearts of fructose‐fed rats (Fig. 1B) and this, in combination with the twofold increase in the half‐maximal inhibitory concentration (IC_50_) of malonyl‐CoA on CPT1 activity (Fig. 1C) indicates that CPT1 activity was overall less inhibited at any malonyl‐CoA concentration in hearts of fructose‐fed rats compared with age‐matched controls. Hence, cardiac mitochondria from fructose‐fed rats experience an increased influx of long‐chain fatty acids. In agreement with these data, mitochondrial oxygen consumption under State 3 respiration (in the presence of ADP) with palmitoyl‐CoA as substrate was higher in hearts of fructose‐fed rats, while it was similar with palmitoylcarnitine (Table 4). State 3 mitochondrial oxygen consumption with octanoylcarnitine as substrate was also higher (Table 4), consistent with increased *β*‐HCAD (*β*‐hydroxyacyl‐CoA dehydrogenase) and MCAD (medium‐chain acyl‐CoA dehydrogenase) activities (Table 2). LCAD (long‐chain acyl‐CoA dehydrogenase) activity was slightly but significantly reduced in hearts of fructose‐fed animals (Table 2), consistent with accumulation of long‐chain acylcarnitines (Table 5).

**Table 4:** Mitochondrial respirometry data

	State 3 (ADP present)			State 4 (Oligomycin present)			(State 3/State 4) Ratio		
	C	FF	*P* value	C	FF	*P* value	C	FF	*P* value
Pyruvate/Malate	19.6 ± 1.9	20.3 ± 1.4	0.781	1.55 (1.37; 2.39)	1.25 (1.18; 1.47)	0.161	11.7 ± 1.1	14.7 ± 1.3	0.0997
Glutamate/Malate	14.9 ± 2.2	13.5 ± 2.5	0.674	1.13 (0.77; 1.93)	1.02 (0.93; 1.51)	0.798	12.0 ± 1.0	11.6 ± 1.9	0.866
Succinate	16.5 ± 2.2	17.8 ± 1.5	0.630	3.03 (2.20; 5.08)	3.29 (2.76; 4.23)	0.721	5.3 (3.9; 5.6)	5.2 (5.1; 5.5)	1.000
Ascorbate/TMPD	35.7 ± 4.1	47.6 ± 4.7	0.079	—	—	—	—	—	—
Pal‐CoA/Car/Malate	5.4 ± 0.8	9.1 ± 0.4	**0.0008**	0.92 ± 0.10	0.94 ± 0.04	0.857	5.9 ± 0.8	9.8 ± 0.4	**0.0007**
PalCar/Malate	6.8 ± 1.2	7.0 ± 0.9	0.875	1.10 ± 0.15	1.13 ± 0.12	0.888	6.5 ± 1.0	6.5 ± 0.8	0.995
OctCar/Malate	7.9 ± 0.6	10.3 ± 0.8	**0.0296**	0.97 (0.85; 1.21)	1.17 (0.95; 1.32)	0.195	7.8 ± 0.7	9.1 ± 0.7	0.174
AcetylCar/Malate	9.5 ± 1.0	11.6 ± 1.3	0.203	0.88 ± 0.11	0.89 ± 0.11	0.981	11.2 ± 1.0	13.6 ± 0.9	0.0961

Data are presented as mean ± SE or median (25th; 75th percentile), expressed in pmol O_2_/(s *u*g) mitochondria (*n* = 7–8/group). Bold indicates the results significant at the 0.05 level.

C, age‐matched control rats (fed standard chow and water); FF, rats fed standard chow and 10% fructose added to the drinking water; Pal‐CoA, palmitoyl‐CoA; PalCar, palmitoylcarnitine; OctCar, octanoylcarnitine; AcetylCar, acetylcarnitine; Car, carnitine; TMPD, tetramethyl‐p‐phenylenediamine.

**Table 5:** Concentrations of carnitine and acylcarnitine species in cardiac tissue of healthy control and fructose‐fed rats

Acylcarnitine species	C	FF	*P* value
Free carnitine [C0]	424 ± 22	492 ± 36	0.122
Acetylcarnitine [C2]	375 ± 40	491 ± 15	0.053
Propionylcarnitine [C3]	1.7 ± 0.2	2.4 ± 0.3	0.064
Butyrylcarnitine [C4]	0.8 ± 0.1	1.1 ± 0.2	0.115
3‐OH‐Butyrylcarnitine/Malonylcarnitine	1.2 ± 0.1	1.9 ± 0.1	**0.005**
Isovalerylcarnitine [C5]	0.44 (0.37; 0.47)	0.57 (0.42; 0.79)	0.114
Tiglylcarnitine [C5:1]	0.11 ± 0.01	0.13 ± 0.02	0.327
Hexanoylcarnitine [C6]	0.78 ± 0.07	0.90 ± 0.11	0.366
Methylmalonylcarnitine [C4‐DC]	3.2 ± 0.3	3.9 ± 0.3	0.217
Glutarylcarnitine [C5‐DC]	0.17 (0.14; 0.19)	0.20 (0.15; 0.26)	0.476
Methylglutarylcarnitine [C6‐DC]	0.05 ± 0.005	0.06 ± 0.008	0.111
Octanoylcarnitine [C8]	0.27 ± 0.01	0.34 ± 0.02	**0.027**
Octenoylcarnitine [C8:1]	0.05 ± 0.01	0.04 ± 0.01	0.455
Decanoylcarnitine [C10]	0.12 ± 0.01	0.19 ± 0.04	0.090
Decenoylcarnitine [C10:1]	0.04 ± 0.01	0.04 ± 0.01	0.911
Lauroylcarnitine [C12]	0.28 ± 0.02	0.41 ± 0.08	0.101
Laureoylcarnitine [C12:1]	0.21 ± 0.02	0.30 ± 0.05	0.061
Myristdienoylcarnitine [C14:2]	0.20 (0.19; 0.24)	0.32 (0.26; 0.61)	**0.019**
Myristeoylcarnitine [C14:1]	0.39 ± 0.02	0.82 ± 0.18	**0.019**
Myristoylcarnitine [C14]	1.04 ± 0.07	1.57 ± 0.26	**0.043**
3‐OH‐Myristoylcarnitine [C14‐OH]	0.29 ± 0.03	0.34 ± 0.02	0.265
3‐OH‐Myristeoylcarnitine [C14:1‐OH]	0.36 ± 0.05	0.53 ± 0.05	0.057
Palmiteoylcarnitine [C16:1]	2.0 (1.9; 2.2)	4.7 (3.0; 5.8)	**0.010**
Palmitoylcarnitine [C16]	6.1 ± 0.4	8.1 ± 1.3	0.110
3‐OH‐Palmitoylcarnitine [C16‐OH]	1.2 ± 0.2	1.5 ± 0.02	0.294
3‐OH‐Palmiteoylcarnitine [C16:1‐OH]	0.89 ± 0.10	1.44 ± 0.04	**0.003**
Linoleoylcarnitine [C18:2]	6.9 (4.7; 8.5)	7.7 (7.1; 15.6)	0.476
Oleoylcarnitine [C18:1]	12.3 ± 0.8	19.0 ± 2.4	**0.014**
Stearoylcarnitine [C18]	1.58 ± 0.09	1.97 ± 0.23	0.100
3‐OH‐Stearoylcarnitine [C18‐OH]	0.21 ± 0.03	0.22 ± 0.02	0.708
3‐OH‐Oleoylcarnitine [C18:1‐OH]	1.93 ± 0.30	2.61 ± 0.23	0.143
Total acylcarnitines	420 ± 41	557 ± 44	**0.037**

Values represent the acylcarnitine content expressed in nmol/g protein (*n* = 6 for C and *n* = 4 for FF). Data are presented as mean ± SE or median (25th percentile; 75th percentile). Bold indicates the results significant at the 0.05 level.

C, age‐matched control rats (fed standard chow and water); FF, rats fed standard chow and 10% fructose added to the drinking water.

### Mitochondrial respiration is preserved despite reduced mitochondrial content in hearts of early type‐2 diabetic fructose‐fed rats

**Figure 2:**
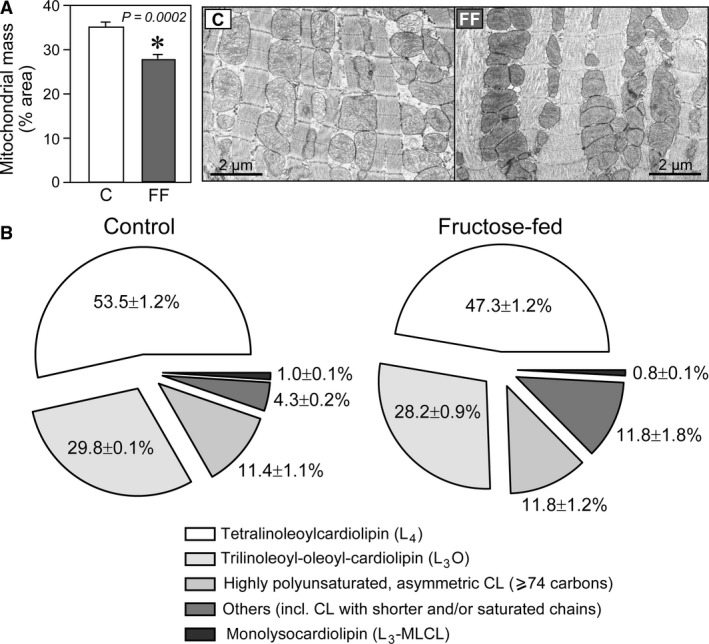
Changes in mitochondrial mass and cardiolipin pattern in hearts of fructose‐fed rats. (A) Reduced mitochondrial mass in hearts of fructose‐fed rats. Representative transmission electron microscopy (TEM) images at 3000× magnification of heart sections and analysis of mitochondrial mass (*n* = 5 at 1500× or 3000× magnification per group). Results are mean ± SE. C, control rats; FF, fructose‐fed rats. (B) Relative amounts of cardiolipin (CL) species in hearts of control and fructose‐fed rats (*n* = 6/group). Data are presented as mean ± SE.

Citrate synthase (CS) activity, a measure of mitochondrial content and oxidative capacity, was significantly reduced in hearts of fructose‐fed rats (Table 2). Moreover, the protein levels of the mitochondrial marker complex IV were also lower in hearts of fructose‐fed rats (Fig. 1A). These data were corroborated by electron microscopy showing a reduced mitochondrial mass in hearts of fructose‐fed rats (Fig. 2A). In contrast, mitochondrial respiratory activity determined during active oxidative phosphorylation (State 3, in the presence of ADP) supported by pyruvate/malate, glutamate/malate, succinate and ascorbate/TMPD were not different between groups, indicative of no change in respiratory capacity per mitochondrial protein mass (Table 4). Oligomycin‐induced State 4 respiration (proton leak or nonphosphorylating respiration) using pyruvate/malate, glutamate/malate, succinate and ascorbate/TMPD, or fatty acid substrates, was not different between hearts of age‐matched control and fructose‐fed rats. There was no indication of enhanced mitochondrial proton leak or uncoupling. The respiratory control ratio (State 3/State 4 ratio), indicative of the coupling efficiency between substrate oxidation and ATP synthesis, was unaffected in hearts of fructose‐fed rats. The respirometric (complex IV‐driven substrate ascorbate/TMPD; Table 4) and the enzymatic assay (Table 2) suggest that cardiac complex IV activity is preserved in hearts of fructose‐fed rats. However, subtle changes in complex I and complex II enzymatic activities were detected (Table 2).

The content of cardiolipin, which is found almost exclusively in the inner mitochondrial membrane, where it is essential for the optimal function of numerous enzymes involved in mitochondrial energy metabolism, was similar in cardiac mitochondria from age‐matched control and fructose‐fed rats (Table 6). However, alterations in cardiolipin composition were observed (Fig. 2B). In particular, cardiolipin species containing saturated fatty acyl chains were increased in hearts of fructose‐fed rats. For example, the cardiolipin cluster with 68 carbons accounted for 0.4% (0.03%; 0.04%) of the total cardiolipin in cardiac mitochondria from age‐matched control rats, but this tended to be higher (0.8% [0.05%; 0.13%], *P* = 0.056) in cardiac mitochondria from fructose‐fed rats. Identification of molecular isoforms revealed that both single cardiolipin species (cardiolipin [68:2] identified as C16:0/C16:0/C18:1/C18:1) or mixtures of different isomers were present, such as cardiolipin (68:5) containing C16:0/C16:1/C18:2/C18:2, C16:1/C16:1/C18:1/C18:2, and C14:0/C18:1/C18:1/C18:2. No oxidized cardiolipins were detected.

**Table 6:** Major (>1% abundance) cardiolipin fatty acyl molecular species detected in mitochondrial membranes from healthy control and fructose‐fed rats

CL(CN:DB)	CL molecular species	C	FF	*P* value
CL(54:6)	C18:2/C18:2/C18:2/0:0 (monolysocardiolipin; L_3_‐MLCL)	0.06 ± 0.002	0.04 ± 0.005	**0.019**
CL(70:6)	C16:1/C18:2/C18:1/C18:2	0.06 (0.04; 0.06)	0.17 (0.10; 0.22)	**0.002**
CL(70:7)	C16:1/C18:2/C18:2/C18:2	0.08 ± 0.01	0.29 ± 0.05	**0.003**
CL(72:7)	C18:2/C18:2/C18:1/C18:2 (tri‐linoleoyl‐oleoyl‐cardiolipin; L_3_O)	1.90 ± 0.14	1.56 ± 0.13	0.113
CL(72:8)	C18:2/C18:2/C18:2/C18:2 (tetralinoleoyl cardiolipin; L_4_)	3.42 ± 0.31	2.60 ± 0.	**0.046**
CL(72:9)	C18:2/C18:3/C18:2/C18:2	0.07 ± 0.004	0.04 ± 0.007	**0.025**
CL(74:8)	C18:1/C18:2/C18:2/C20:3; C18:1/C18:2/C18:1/C20:4	0.11 (0.11; 0.13)	0.13 (0.08; 0.16)	0.937
CL(74:9)	C18:2/C18:2/C18:2/C20:3 (major); C18:1/C18:2/C18:2/C20:4	0.19 (0.01)	0.22 (0.02)	0.279
CL(74:10)	C18:2/C18:3/C18:2/C20:3; C18:2/C18:2/C18:2/C20:4; C18:2/C20:4/C18:2/C22:6	0.13 (0.12; 0.16)	0.10 (0.08; 0.13)	0.065
CL(76:11)	C18:1/C18:2/C18:2/C22:6; C16:0/C18:2/C20:4/C22:5; C16:0/C18:2/C20:3/C22:6	0.11 (0.10; 0.15)	0.09 (0.05; 0.11)	0.093
CL(76:12)	C18:2/C18:2/C18:2/C22:6 (major); C18:1/C18:1/C20:4/C22:6	0.12 (0.11; 0.18)	0.07 (0.03; 0.11)	**0.026**
Total content	All species, including low abundance species	6.16 ± 0.42	5.49 ± 0.38	0.268

Values represent the molecular species content expressed in nmol/mg protein and are presented as mean ± SE or median (25th; 75th percentile). *n* = 6 hearts per group. Bold indicates the results significant at the 0.05 level.

C, age‐matched control rats (fed standard chow and water); FF, rats fed standard chow and 10% fructose added to the drinking water; CL, cardiolipin; CN, carbon number of all 4 acyl chains; DB, number of double bonds on all 4 acyl chains.

### Evidence of oxidative stress in hearts of early type‐2 diabetic fructose‐fed rats

**Figure 3:**
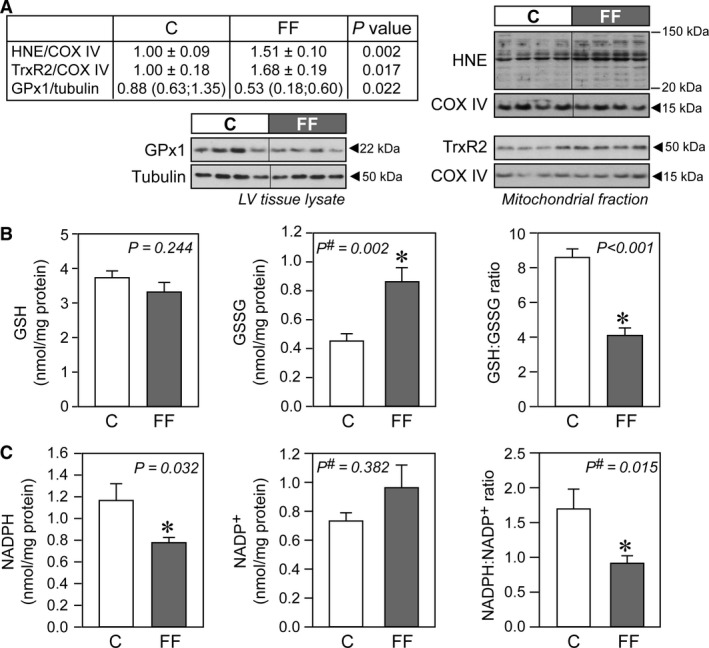
Increased oxidative stress in hearts of fructose‐fed rats. (A) Representative immunoblots and densitometric quantification of mitochondrial 4‐hydroxynonenal (HNE), mitochondrial thioredoxin reductase (TrxR2), and cellular glutathione peroxidase 1 (GPx1) (*n* = 6–7/group). (B) Concentrations of reduced glutathione (GSH) and oxidized glutathione (GSSG) in cardiac homogenates of control and fructose‐fed rats, with their respective GSH:GSSG ratio (*n* = 10/group). (C) Concentrations of NADPH and NADP ^+^ in cardiac mitochondria of control and fructose‐fed rats, with their respective NADPH/NADP ^+^ ratio (*n* = 8/group). Data are presented as mean ± SE. ^#^Mann–Whitney nonparametric statistical test was applied. C, control rats; FF, fructose‐fed rats.

Mitochondrial 4‐hydroxynonenal (HNE), which constitutes one of the most reactive aldehydes that are generated within a pathological milieu such as diabetes (Roede and Jones 2010), was higher in hearts of fructose‐fed rats (Fig. 3A). In addition, the activity of ALDH2, a key enzyme involved in HNE metabolism and a cellular defense mechanism against HNE toxicity, was reduced in hearts of fructose‐fed rats (Table 2) despite similar protein levels (data not shown). Accordingly, the activity of mitochondrial aconitase (a TCA cycle enzyme susceptible to oxidative stress) was lower in hearts of fructose‐fed rats (Table 2). Ratios of GSH:GSSG (Fig. 3B) and NADPH:NADP^+^ (Fig. 3C), key indices of the redox status, were reduced. Protein levels of glutathione peroxidase (GPx1) were lower and those of thioredoxin reductase (TrxR2) were higher in hearts from fructose‐fed rats (Fig. 3A). The activities of mitochondrial and cytosolic SOD were unchanged (Table 2).

### Sirtuin expression and activity in hearts of early type‐2 diabetic fructose‐fed rats

**Figure 4:**
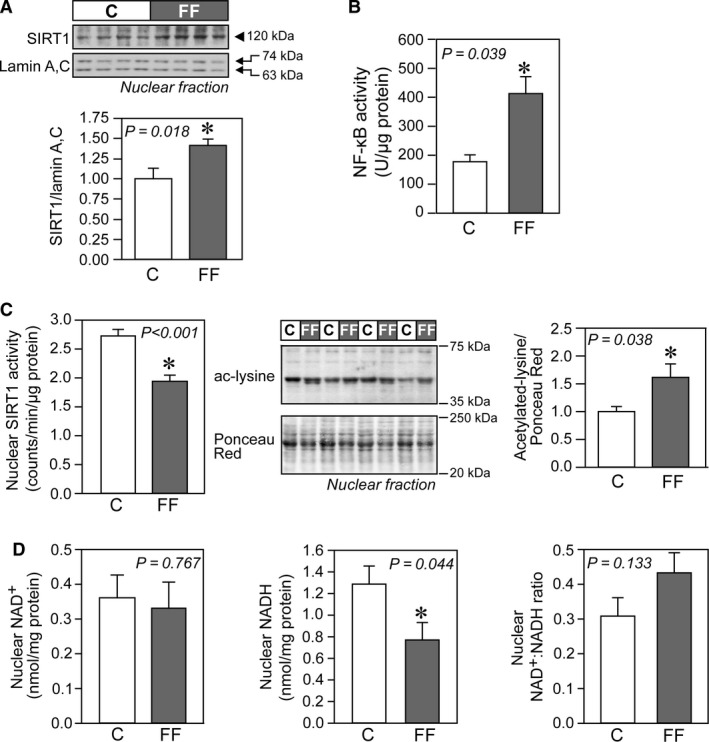
Sirtuin 1 (SIRT1) signaling in hearts of fructose‐fed rats. (A) Representative immunoblot and densitometric analysis of nuclear SIRT1 level (*n* = 7/group). (B) Nuclear factor‐*κ*B (NF‐*κ*B) DNA‐binding activity in cardiac tissues of control and fructose‐fed rats, measured from nuclear extracts (*n* = 6/group). (C) Nuclear SIRT1 activity and global acetylation profile of nuclear proteins (*n* = 6/group). The entire range (20–250 kDa) of Ponceau Red‐stained proteins was analyzed and used to normalize acetylated proteins (between 35 and 75 kDa) in respective lanes. (D) Nuclear concentrations of NAD ^+^ and NADH in cardiac tissues of control and fructose‐fed rats, with their respective NAD ^+^/NADH ratio (*n* = 8/group). Results are mean ± SE. ^#^Mann–Whitney nonparametric statistical test was applied. C, control rats; FF, fructose‐fed rats.

**Figure 5:**
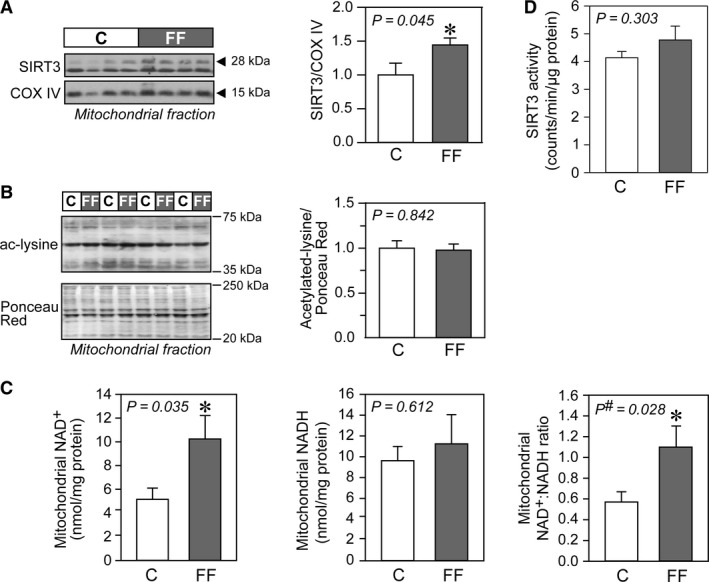
Sirtuin 3 (SIRT3) signaling in hearts of fructose‐fed rats. Representative immunoblots and densitometric analyses of (A) mitochondrial SIRT3 level (*n* = 7/group) and (B) global acetylation profile of mitochondrial proteins (*n* = 6/group). The entire range (20–250 kDa) of Ponceau Red‐stained proteins was analyzed and used to normalize acetylated proteins (between 35 and 75 kDa) in respective lanes. (C) Mitochondrial concentrations of NAD ^+^ and NADH in cardiac tissues of control and fructose‐fed rats, with their respective NAD ^+^/NADH ratio (*n* = 8/group). (D) Mitochondrial SIRT3 activity (*n* = 6/group). Results are mean ± SE. C, control rats; FF, fructose‐fed rats.

In hearts of fructose‐fed rats, nuclear SIRT1 protein levels were increased (Fig. 4A) but SIRT1 activity was reduced as compared to control hearts (Fig. 4C). The reduced SIRT1 activity was mirrored in the observed increase in global nuclear protein acetylation (Fig. 4C). Reduced activity of SIRT1 is accompanied by NF‐*κ*B activation (Fig. 4B) and may drive inflammation in hearts from fructose‐fed rats. Finally, there was no difference in the NAD^+^/NADH ratio (an important determinant of sirtuin activity) in the nuclear fraction (Fig. 4D). A similar dissociation between protein abundance and activity levels was also observed with regard to mitochondrial SIRT3. Despite a doubling in SIRT3 protein abundance (Fig. 5A) and NAD^+^/NADH ratio (Fig. 5C) in hearts of fructose‐fed rats, SIRT3 activity within the mitochondrial fraction was unchanged compared with hearts from age‐matched rats (Fig. 5D). The lack of SIRT3 activation was corroborated by the absence of changes in global mitochondrial protein acetylation (Fig. 5B).

## Discussion

In this study, we investigated the early molecular events associated with cardiac insulin resistance in the fructose‐induced T2DM rat model. Furthermore, we studied whether fructose feeding for 6 weeks in the rat would lead to cardiac mitochondrial dysfunction as previously reported in other animal models (Boudina and Abel 2010; Buchanan et al. 2005). Our data show a clear pattern of altered substrate utilization characterized by (1) reduced PDH activity (2) increased sarcolemmal (CD36) and mitochondrial fatty acid uptake (CPT1B), (3) increased State 3 respiration with fatty acid substrates, (4) increased MCAD and *β*‐HCAD activities, and (5) increased nuclear expression of PPAR*α* and PGC1*α*, all consistent with predominant reliance of cardiac metabolism on fatty acid utilization.

Over‐reliance on fatty acid oxidation in the diabetic heart has been consistently observed in many experimental models of T2DM (Boudina and Abel 2010; Buchanan et al. 2005; Carley and Severson 2005; Mazumder et al. 2004; Wang et al. 2005, 2015) and has been linked to the activation of PPAR*α* and its coactivator PGC1*α* (Duncan et al. 2007; Finck et al. 2002; Wright et al. 2009). The upregulation of PPAR*α* and PGC1*α* seen in our study confirms observations by Duncan et al. (2007) and drives the transcription of genes involved in fatty acid metabolism (such as CD36, CPT1, MCAD) in order to cope with excess provision of lipids from the circulation, and of PDK4 (Wende et al. 2005), which further suppresses glucose oxidation by decreasing PDH activity (Wu et al. 1998). PDH is an important metabolic control point where the competition between carbohydrates and fatty acids as metabolic substrates is rapidly regulated in the heart (Lopaschuk et al. 2010). It has been recently recognized that the increase in PDK4 protein levels is extremely rapid and occurs within the first day when feeding mice a high‐fat diet (Crewe et al. 2013, 2017). Likewise, the turnover of malonyl‐CoA, the allosteric inhibitor of CPT1, is very rapid in the heart (Reszko et al. 2001), given the high expression of acetyl‐CoA carboxylase (responsible for its synthesis) and malonyl‐CoA decarboxylase (responsible for its degradation). In addition, high levels of circulating fatty acids may be also responsible for the increased activity of AMPK, which in turn inhibits acetyl‐CoA carboxylase, leading to reduced malonyl‐CoA synthesis as originally reported by Clark et al. (2004). Alternatively, the observed reduced malonyl‐CoA levels could be explained by increased activity of malonyl‐CoA decarboxylase, which is a known PPAR*α* target (Campbell et al. 2002) and has been reported to be responsible for the increased fatty acid utilization in type‐1 diabetic hearts (Sakamoto et al. 2000).

Cardiac insulin resistance (reduced glucose uptake, blunted Akt‐mediated insulin signaling, AMPK activation) is one of the earliest observed defects in mice fed with high‐fat diet and develops after only 10 days, well in advance of other metabolic organs such as skeletal muscle, adipose tissue, and liver (Park et al. 2005). As in genetic models of type‐2 diabetes (*ob/ob* and *db/db* mice), PPAR*α* activation and related changes in expression levels of PPAR*α* targets such as PDK4 or MCAD appear only after 5 weeks (Buchanan et al. 2005; Wright et al. 2009). Hence, the molecular changes observed in hearts from rats fed fructose‐enriched water for 6 weeks suggest that the initial stage of “feedforward” activation of *β*‐oxidation has been replaced by a chronic PPAR*α*‐driven increase in flux through this pathway.

Overall mitochondrial oxidative phosphorylation per mitochondrial mass was not compromised by fructose feeding. Moreover, respiration was increased when using fatty acids substrates, indicating enhanced fatty acid oxidation. In humans, long‐standing diabetes has been associated with mitochondrial dysfunction (Anderson et al. 2009a; Croston et al. 2014; Montaigne et al. 2014), but animal models yielded conflicting findings. Kuo et al. (1985) showed impaired state 3 respiration of mitochondria isolated from type‐2 diabetic *db/db* mouse hearts oxidizing pyruvate, a defect that was linked to decreased PDH activity. The authors further showed that there was no difference in respiration, when using palmitoylcarnitine as substrate and the activity of both *β*‐hydroxyacyl‐CoA dehydrogenase and of *β*‐ketothiolase (*α* and *β* subunit of the mitochondrial trifunctional protein, respectively) were not altered by diabetes. Likewise, reduced mitochondrial respiration has been reported both in *db/db* mouse hearts (Boudina et al. 2007; Tocchetti et al. 2012) and *ob/ob* mouse hearts (Boudina et al. 2005). On the other hand, a recently published comprehensive longitudinal metabolomics analysis on the interaction between age and high‐fat diet in the *ob/ob* mouse heart revealed that mitochondrial function is largely preserved in *ob/ob* mice fed with conventional rodent diet and defects in both complex I and complex II only appeared as the mice aged (10 months) (Wang et al. 2015). Switching to a high‐fat diet lead to a significant increase in respiratory capacity when oxidizing fatty acids after 3 and 12 weeks (Wang et al. 2015).

While mitochondrial respiration in the presence of substrates feeding electrons into the electron transport system was unchanged when expressed per mitochondrial mass, the total oxidative phosphorylation in cardiac tissue is also dependent on the mitochondrial content. Here, we used several markers to estimate mitochondrial density, namely CS activity, COX IV protein levels, and 2D TEM analysis. All three methods showed that mitochondrial content is reduced in early type‐2 diabetic hearts of fructose‐fed rats. Morphometric studies of cardiac mitochondria in animal models of type‐2 diabetes are very limited. A mitochondrial biogenic response (increased number, but somewhat smaller mitochondria) has been reported to occur in the *db/db* mouse heart aged 8–9 weeks (Boudina et al. 2007). However, at 18 weeks of age decreased mitochondrial size as well as decreased state 3 respiration rates were reported for subsarcolemmal mitochondria (Dabkowski et al. 2010). In *ob/ob* mice aged 12 weeks a qualitative examination revealed mitochondrial swelling and cristae disorganization (Dong et al. 2006). Mitochondrial content and size reflect the interplay between mitophagy (i.e., the degradation of existing, potentially damaged, mitochondria), biogenesis, and fusion/fission events. Generally speaking, mitochondria can quickly adapt to changes in metabolic flux (Eisner et al. 2017), but clearly much more work is necessary to map morphological changes and fusion/fission events in diabetic mitochondria.

Our experiments also revealed fatty acid remodeling in cardiolipin. Cardiolipin is a membrane phospholipid found predominantly in the inner mitochondrial membrane. The symmetric form (tetralinoleoyl‐CL or L_4_‐CL) is the most abundant in the mammalian heart (Schlame et al. 2005). It is essential for optimal activities of individual respiratory system complexes and supercomplexes, of ATP synthase and adenine nucleotide translocase (Paradies et al. 1837). Cardiolipin content and remodeling changes the overall efficiency of the respiratory system (Vergeade et al. 2016; Ye et al. 2016). In our study, we observed alterations in cardiac cardiolipin fatty‐acid species composition, namely a loss of the symmetric tetralinoleoyl‐CL in favor of more asymmetric cardiolipins with shorter and more saturated chains (Fig. 2B, Table 6). Similar cardiolipin abnormalities have been previously reported to occur in *ob/ob* mouse hearts aged 12–16 weeks (Han et al. 2007), however, the causes and the bioenergetic consequences of these changes are largely unknown and warrant further investigation.

Increased mitochondrial fatty acid oxidation exerts pressure on the respiratory chain to reduce increasing amounts of NADH and FADH_2_, leading to enhanced ROS production (Fisher‐Wellman and Neufer 2012; Rindler et al. 2013). The following mechanisms are likely responsible for the oxidative stress observed in hearts of fructose‐fed rats. First, superoxide production may increase as a result of an enhanced complex I activity in the absence of any corresponding increase in downstream complex III activity. Second, excess in reducing equivalents (i.e., NADH and FADH_2_) provided by enhanced fatty acid oxidation increase the QH_2_/Q ratio, which consequently increases ROS production by (1) limiting the availability of oxidized Q as an electron acceptor for complex I, (2) generating reverse electron transport back into complex I, and/or (3) increasing the electron pressure at complex III (Fisher‐Wellman and Neufer 2012; Rindler et al. 2013). This fatty acid oxidation‐dependent ROS generation may be a hallmark of T2DM and has been reported to occur in the heart (Boudina et al. 2007; Wang et al. 2015), skeletal muscle (Anderson et al. 2009b; Seifert et al. 2010), and kidney (Rosca et al. 2012).

A reduced efficiency of ROS scavenging systems results in a more oxidizing intracellular environment. Indeed, the activity of the redox‐sensitive TCA cycle enzyme aconitase was reduced and we observed increased HNE‐modified mitochondrial proteins in hearts of fructose‐fed rats. Furthermore, both the GSH/GSSG ratio and NADPH:NADP^+^ ratio were significantly reduced. Increased cardiac oxidative stress has been observed in *ob/ob* mice fed a high‐fat diet (reduced GSH/GSSG ratio) (Wang et al. 2015) and in *db/db* mice (increased HNE‐modified proteins and increased malondialdehyde levels) (Boudina et al. 2007). It is important to note that in the healthy heart, HNE ‐an aldehyde derivative generated from lipid peroxidation‐ is readily metabolized by conjugation to glutathione (and subsequently removed by aldose reductase) or by oxidation to 4‐hydroxynonenoic acid by aldehyde dehydrogenase (Srivastava et al. 1998). Accumulation of HNE‐protein adducts is therefore an indicator of reduced glutathione availability, reduced aldehyde dehydrogenase content, or depletion of NAD^+^, an important cofactor determining aldehyde dehydrogenase activity. The activity of ALDH2 (mitochondrial isoform) was significantly reduced in cardiac mitochondria from fructose‐fed rats as compared to age‐matched healthy rats, consistent with increased formation of HNE‐adducts. The reasons for the impaired activity of ALDH2 are not clear, but it has been recently reported that HNE forms adducts with ALDH2 itself and attenuates its activity (Mali et al. 2014). Surprisingly, mitochondrial NAD^+^ levels and the NAD^+^/NADH ratio were higher in hearts from fructose‐fed rats. However, the method used to measure these metabolites cannot distinguish between protein‐bound and free NAD(H) and may not reflect the availability of free NAD^+^.

Our results further suggest that the antioxidant response in hearts of fructose‐fed rats is perturbed, as evidenced by the unchanged activity of both MnSOD (the primary mitochondrial antioxidant enzyme) and cytoplasmic CuZnSOD, both of which are responsible for the dismutation of superoxide to H_2_O_2_. This occurs despite the observed increase in NF‐*κ*B activity, known to regulate the expression of several antioxidant proteins, including MnSOD (reviewed in Morgan and Liu 2011). H_2_O_2_ can alter the redox state of the cell by either reacting directly with thiol residues within redox‐sensitive proteins or by shifting the GSH/GSSG ratio (Schafer and Buettner 2001). Detoxification of H_2_O_2_ is achieved enzymatically by glutathione peroxidases, catalase, and peroxiredoxins. We found reduced abundance of GPx‐1 that uses GSH as an obligate cosubstrate in the reduction in H_2_O_2_ to water and is the most important peroxidase for cellular H_2_O_2_ removal. In the heart, it is also a key enzyme responsible for the removal of intramitochondrial peroxides (Antunes et al. 2002). The thioredoxin‐2 (Trx2) and GSH systems function in parallel in peroxide metabolism (Zhang et al. 2007). The increased levels of TrxR2 (a central component of the thioredoxin‐2 system) may be thus interpreted as a compensatory redox adaptive response, given that the disulfide in the thioredoxin active site is reduced by TrxR2 at the expense of NADPH, the levels of which are significantly reduced in hearts from fructose‐fed rats. Increased protein levels of cardiac TrxR2 have been previously observed in mice on a high‐fat, high‐sugar diet (Fisher‐Wellman et al. 2013). In that study, the authors also uncovered the critical role played by this enzyme in suppressing mitochondrial H_2_O_2_ emission, specifically during fatty acid oxidation (Fisher‐Wellman et al. 2013).

Interestingly, our study demonstrates that nuclear SIRT1 and mitochondrial SIRT3 protein levels (both increased in cardiac tissue of fructose‐fed rats) do not adequately explain their respective changes in activity (reduced in the case of SIRT1 and unaffected, in the case of SIRT3). Activation of PPAR*α* can regulate SIRT1 expression in a positive manner (Hayashida et al. 2010). In addition, an intricate crosstalk exists between NF‐*κ*B and SIRT1 signaling (Kauppinen et al. 2013) affecting protein levels. For instance, the expression of SIRT1 may increase in NF‐*κ*B‐driven inflammation (Zhang et al. 2010). In the heart, a moderate increase in SIRT1 may serve to enhance the tolerance to oxidative stress, as originally reported by Alcendor et al. (2007). On the other hand, SIRT1 activation can inhibit NF‐*κ*B signaling either directly by deacetylating the RelA/p65 subunit (Yeung et al. 2004) or indirectly at the transcriptional level (Pfluger et al. 2008). SIRT1 activity may be regulated directly or indirectly by a number of different factors such as (Aguilar‐Arnal et al. 2016) changes in free NAD^+^, (Alcendor et al. 2007) levels of the inhibitor molecule nicotinamide (product inhibition), (Alrob et al. 2014) flux through the nicotinamide salvage pathway, (Anderson et al. 2009a) oxidative modification of key residues required for catalysis. Very little is known on the temporal evolution of SIRT1 activity in the development and progression of cardiac insulin resistance. Sulaiman et al. (2010) studied the temporal pattern of SIRT1 activity in hearts of streptozotocin (STZ)‐induced type‐1 diabetic in mice. Cardiac SIRT1 protein levels were found to be significantly increased in the heart of diabetic mice 1 month after STZ administration without any changes in SIRT1 deacetylase activity. However, protein levels were significantly reduced and activity severely compromised in advanced type‐1 diabetes (3 months following STZ injection) (Sulaiman et al. 2010). We speculate that increased protein levels of SIRT1 in fructose‐fed rats may represent an adaptive response to promote tolerance against oxidative stress. The concomitant decline in activity could be the result of a decline in free NAD^+^ (as the method used to measure NADH and NAD^+^ cannot distinguish between free and protein‐bound nucleotides) or could be due to oxidative posttranslational modifications. Indeed, there are studies indicating that the cysteine residues in SIRT1 are vulnerable to oxidative and nitrosative stress, which lead to impaired sirtuin activity (Caito et al. 2010; Zee et al. 2010; Shao et al. 2014).

Of the NAD‐dependent lysine deacetylases present in mitochondria (SIRT3, SIRT4, and SIRT5), only SIRT3 appears to regulate mitochondrial protein acetylation levels (Hirschey et al. 2011; Bugger et al. 2016). Our results show increased SIRT3 protein levels in cardiac mitochondria from fructose‐fed rat as compared to rats fed a standard diet. In the study by Hirschey and co‐workers (Hirschey et al. 2011), feeding mice a high‐fat diet for 13 weeks reduced liver SIRT3 protein content and significantly increased hepatic mitochondrial protein acetylation as compared to mice fed standard diet. The authors attributed the loss of SIRT3 protein to the reduction in PGC‐1*α*, a known regulator of SIRT3 (Kong et al. 2010). A similar reduction in SIRT3 levels has been observed in the heart (Alrob et al. 2014; Zeng et al. 2015), although the authors of those studies did not investigate potential mechanisms. Moreover, there may have been a biasing interaction between chronic high‐fat feeding and aging in the mentioned studies (Kwon et al. 2015). It is interesting to note that chronic caloric restriction results in a dramatically different and tissue‐dependent regulation of mitochondrial acetylation (Schwer et al. 2009). The authors found that protein acetylation was significantly increased in liver mitochondria (despite a moderate increase in SIRT3 protein levels), significantly decreased in brown adipose tissue, but almost unaffected in heart, kidney, and brain (Schwer et al. 2009). In our study (short‐term fructose feeding), we think that the increased cardiac SIRT3 protein levels may be explained by increased activity of PGC‐1*α*. Despite increased protein levels, SIRT3 deacetylase activity was unaffected by fructose feeding and the acetylation pattern of mitochondrial proteins was similar in hearts from fructose‐fed rats as compared to healthy age‐matched rats. We speculate that the increased protein levels may represent an adaptive response to either a decrease in free NAD^+^, despite increased activity of complex I and related regeneration of NAD^+^ (the total levels of which are increased in cardiac mitochondria from fructose‐fed rats), or SIRT3 carbonylation, which has been shown to negatively affect its activity (Fritz et al. 2011). Indeed, sirtuins are important in facilitating metabolic adaptations to nutritional stress (caloric restriction and overnutrition). However, our data clearly indicate that sirtuin protein abundance does not necessarily mirror activity and that more research is needed to really dissect the regulatory pathways leading to changes in protein acetylation and function both in health and disease. Novel tools that allow more accurate measurements of free NAD^+^ concentrations in different cellular compartments have been recently become available and indeed reveal the complexities of NAD^+^ signaling (Aguilar‐Arnal et al. 2016; Cambronne et al. 2016).

In conclusion, our study supports the important role of SIRT1‐PPAR*α* signaling in cardiac metabolic adaptations in early T2DM by modulating the fatty acid oxidation pathway. The observed decreased activity of cardiac SIRT1 after 6 weeks of fructose feeding may indicate that the heart is no longer capable to cope with the chronic increased availability of lipids. This may represent one of the earliest events in the development of biochemical derangements that will ultimately lead to inflammation and remodeling in the diabetic heart.

## Conflict of Interest

The authors have nothing to disclose and no author has any conflict of interest.
